# Engineering BinB Pore-Forming Toxin for Selective Killing of Breast Cancer Cells

**DOI:** 10.3390/toxins15040297

**Published:** 2023-04-18

**Authors:** Tipaporn Kumkoon, Chalongrat Noree, Panadda Boonserm

**Affiliations:** Institute of Molecular Biosciences, Mahidol University, Salaya, Phuttamonthon, Nakhon Pathom 73170, Thailand; tipapornkumkoon@gmail.com (T.K.); chalongrat.nor@mahidol.ac.th (C.N.)

**Keywords:** pore-forming toxin, *Lysinibacillus sphaericus*, cell-targeting peptide, breast cancer cells, cell death

## Abstract

Breast cancer is one of the most common cancers in women worldwide. Conventional cancer chemotherapy always has adverse side effects on the patient’s healthy tissues. Consequently, combining pore-forming toxins with cell-targeting peptides (CTPs) is a promising anticancer strategy for selectively destroying cancer cells. Here, we aim to improve the target specificity of the BinB toxin produced from *Lysinibacillus sphaericus* (Ls) by fusing a luteinizing hormone-releasing hormone (LHRH) peptide to its pore-forming domain (BinB_C_) to target MCF-7 breast cancer cells as opposed to human fibroblast cells (Hs68). The results showed that LHRH-BinB_C_ inhibited MCF-7 cell proliferation in a dose-dependent manner while leaving Hs68 cells unaffected. BinB_C_, at any concentration tested, did not affect the proliferation of MCF-7 or Hs68 cells. In addition, the LHRH-BinB_C_ toxin caused the efflux of the cytoplasmic enzyme lactate dehydrogenase (LDH), demonstrating the efficacy of the LHRH peptide in directing the BinB_C_ toxin to damage the plasma membranes of MCF-7 cancer cells. LHRH-BinB_C_ also caused MCF-7 cell apoptosis by activating caspase-8. In addition, LHRH-BinB_C_ was predominantly observed on the cell surface of MCF-7 and Hs68 cells, without colocalization with mitochondria. Overall, our findings suggest that LHRH-BinB_C_ could be investigated further as a potential cancer therapeutic agent.

## 1. Introduction

Breast cancer is the most prevalent cancer among women worldwide. Several cancer treatments, such as chemotherapy, radiation, and immunotherapy, can cause adverse side effects on healthy tissues. This has encouraged the development of novel cancer therapeutic agents that specifically target cancer cells and lower the unwanted effects on healthy cells [1]. Bacterial toxins can efficiently kill cells. Thus, many of them have been studied as potential anticancer agents [2]. The aerolysin-like toxin family is a major class of β pore-forming toxins (β-PFTs) that disrupts the cell membranes, subsequently leading to leakage and death of target cells [3]. Most of them have a variable membrane-binding (β-trefoil) domain and a conserved aerolysin-like pore-forming domain with a similar mechanism of action involving β-barrel structures formed by the assembly of β-hairpins. One kind of aerolysin pore-forming toxins, β-PFT-type parasporins, also known as MTX-like Cry toxins, possess specific cytotoxicity against several types of human cancer cells via specific receptor recognition on the plasma membrane of susceptible cells [4], thereby supporting their application as cancer therapeutic agents. Another aerolysin-type pore-forming toxin known as lamprey immune protein-1 (LIP-1) from *Lampetra japonica* can kill various human cancer cells with minimal effects on normal cells, giving its potential for the application for targeted cancer therapy and early diagnosis [5]. Aerolysin produced by *Aeromonas hydrophilia* can be made specifically for a cancer cell by adding a specific binding moiety such as interleukin 2 (IL2) to selectively kill cancer cells displaying IL2 receptors [6]. Thus, aerolysin-type pore-forming toxins offer great potential for targeting cancer cells, as they not only kill cancer cells selectively but can also be rendered cancer cell-specific by incorporating a targeting agent.

Binary (Bin) toxin produced by *Lysinibacillus sphaericus* (Ls) is composed of BinA (42 kDa) and BinB (51 kDa) that act together to intoxicate *Culex* and *Anopheles* mosquito larvae [7,8]. Both BinA and BinB proteins share structural features in common with those of parasporin-2 (PS2) as well as aerolysin-type pore-forming toxins, suggesting that they may share a common pore-forming mechanism on target cells [9,10,11]. Although aerolysin-type pore-forming toxins share a similar mode of pore formation, they differ in the receptor, number of monomers forming oligomers, pore size, and cellular responses [12].

The previous study demonstrated that the truncated form of BinB containing only the C-terminal or pore-forming domain (referred to as ‘BinB_C_’) exhibited more excellent membrane permeability and membrane perturbation than those of full-length BinB, indicating that this domain plays an essential role in membrane interaction and pore formation [13]. The structural similarity between Bin toxin and parasporin-2 (PS2) suggests that Bin toxin may be cytotoxic to human cancer cells in addition to its mosquitocidal activity. Recent studies have explored the possible cytotoxic activity of Bin toxin towards cancer cells and showed that high concentrations of trypsin-activated Bin toxin (particularly BinB subunit) inhibited cell proliferation of human cancer cell lines, including A549, Caco-2, HK-1, KKU-M055, and HepG2 cells by inducing cell apoptosis, suggesting Bin toxin as a promising candidate for cancer therapy [14,15].

Although using pore-forming toxins could be a promising strategy for cancer therapy, their lack of specificity to cancer cells remains a major problem. To overcome this obstacle, cell-targeting peptides (CTPs) have been used to deliver several anticancer agents to targeted cancer cells through specific binding with their target membrane proteins or receptors that are notably overexpressed on several types of cancer cells to promote their growth and survival [16,17]. LHRH (Luteinizing hormone-releasing hormone), also known as GnRH (Gonadotropin-releasing hormone), is a decapeptide (QHWSYGLRPG) produced by the hypothalamus, has been used to deliver various anti-cancer agents or drugs to cancer cells by using the principle that LHRH peptide will specifically bind to cancer cells with LHRH-receptor being abundant on their cell membrane rather than to the normal cells with low or no expression of LHRH-receptor [18,19,20]. Here, we aim to enhance the breast cancer cell selectivity of a pore-forming or C-terminal BinB domain (BinB_C_) by fusing it with luteinizing hormone-releasing hormone (LHRH) peptide, a targeting molecule for the LHRH receptor on the surface of breast cancer cells. MCF-7 (breast adenocarcinoma) and Hs68 (human foreskin fibroblast) cell lines were used to investigate the specificity and cytotoxicity. The engineered LHRH-BinB_C_ toxin has selective cytotoxicity against LHRH-positive cells in vitro (MCF-7 cells) and may induce cell death via apoptosis, making it a potential alternative treatment for breast cancer.

## 2. Results

### 2.1. Expression and Purification of BinB_C_ and LHRH-BinB_C_ Proteins

**Figure 1:**
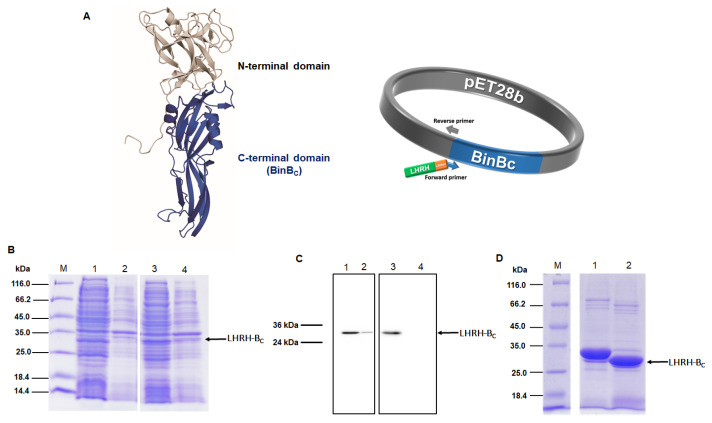
Construction and expression of recombinant BinB_C_ and LHRH-BinB_C_. Construction of recombinant plasmid encoding LHRH-BinB_C_ (**A**) LHRH peptide (green), GSG linker (orange), BinB_C_-encoding gene (blue). The fusion gene is under the control of the T7 promoter and terminator. SDS-PAGE analysis (Coomassie Brilliant Blue R-250 stained gel) and western blotting (using HRP-conjugated anti-polyhistidine antibody [HIS-1] at a 1:5000 ratio) of BinB_C_ and His-LHRH-BinB_C_ expressed in *E. coli* strain BL21(DE3)pLysS strain (**B**,**C**) Lane M, unstained Protein MW Marker. Lanes 1 and 3, soluble fractions after cell lysis of BinB_C_ and His-LHRH-BinB_C_, respectively. Lanes 2 and 4, insoluble fractions after cell lysis of BinB_C_ and His-LHRH-BinB_C_, respectively. SDS-PAGE analysis of LHRH-BinB_C_ protein after removing His_6_-tag by thrombin digestion (**D**) Lane 1, His-LHRH-BinB_C_ (before thrombin digestion), and lane 2, LHRH-BinB_C_ (after thrombin digestion).

The pore-forming domain of BinB protein (BinB_C_), corresponding to residues I201 to Q448, was engineered by adding a luteinizing hormone-releasing hormone (LHRH) targeting peptide (QHWSYGLRPG) to its N-terminus via a flexible glycine-serine linker (GSG) (Figure 1A). The construct was expressed in *E. coli* BL21(DE3) pLysS strain as the N-terminal His_6_-tagged fusion protein (His-LHRH-BinB_C_). BinB_C_ alone without LHRH was also expressed as a His_6_-tagged fusion protein [13], and both forms of proteins were analyzed using 12% SDS-PAGE and western blotting. The BinB_C_ protein was highly expressed as a soluble form with a molecular mass of about 28 kDa, although a small amount of this protein was also detected as an insoluble form (Figure 1B,C). His-LHRH-BinB_C_ protein was predominantly produced as a soluble protein with a molecular mass of about 30 kDa, as predicted (Figure 1B,C). After purification using Ni^2+^-NTA affinity chromatography, the His_6_-tag was removed from the N-terminus of LHRH-BinB_C_ by thrombin digestion to avoid any interference with the LHRH ligand-receptor binding. The thrombin-digested protein had a molecular mass lower by 2 kDa than His-LHRH-BinB_C_. After removing the cleaved His_6_-tag by passing through the Ni^2+^-NTA affinity chromatography followed by gel-filtration chromatography, the LHRH-BinB_C_ with the expected molecular size (~28 kDa) (Figure 1D) was used for further analyses.

### 2.2. LHRH-BinB_C_ Has Specific Cytotoxicity against LHRH-Positive (MCF-7) Cells

**Figure 2:**
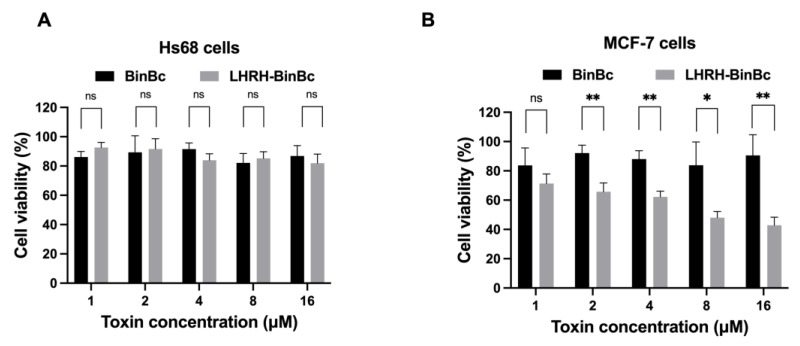
Cell viability (%) of Hs68 (**A**) and MCF-7 (**B**) cells after being treated with BinB_C_ and LHRH-BinB_C_ at different concentrations. After 48 h of incubation with various concentrations of BinB_C_ and LHRH-BinB_C_, the percentage of cell viability in Hs68 (**A**) and MCF-7 (**B**) cells was determined by MTT assay by comparing the absorbance of each sample to that of the negative control (Tris-HCl pH 9.0). All data are presented as mean ± SD from the triplicate analyses. Statistical significance was analyzed using the *t*-test and annotated as follows: ** *p* ≤ 0.01, * *p* ≤ 0.05; ns, not significant (*p* > 0.05).

To examine the cytotoxic effects of engineered LHRH-BinB_C_ compared to those of BinB_C_ on MCF-7 and Hs68 cells, the cells were exposed to BinB_C_ and LHRH-BinB_C_ toxins at concentrations ranging from 1–16 μM, while Tris-HCl pH 9.0 buffer served as a negative control. After observing the morphologies of MCF-7 and Hs68 cells treated with varying toxin concentrations, we found that neither BinB_C_ nor LHRH-BinB_C_ significantly affected the morphology of MCF-7 and Hs68 cells. The results of the MTT assay demonstrated that BinB_C_ at all tested concentrations had no toxic effects on both Hs68 and MCF-7 cells (Figure 2A,B). Hs68 cells showed no change in cell viability after treatment with LHRH-BinB_C_ up to the maximum tested concentration (16 μM) (Figure 2A). On the other hand, LHRH-BinB_C_ could inhibit the proliferation of MCF-7 cells in a dose-dependent manner (Figure 2B), giving an IC_50_ of 10.96 μM. As a negative control, Tris-HCl pH 9 buffer at 16 μM showed no effect on either MCF-7 and Hs68 cells.

### 2.3. LHRH-BinB_C_ Causes Plasma Membrane Damage in MCF-7 Cells

**Figure 3:**
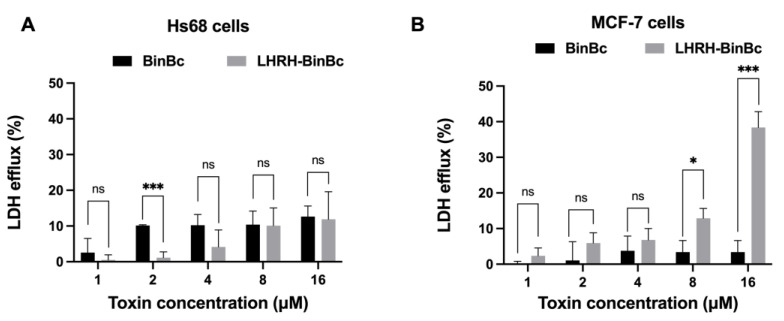
Effects of BinB_C_ and LHRH-BinB_C_ on plasma membrane permeability of Hs68 and MCF-7 cells. After incubation with various concentrations of BinB_C_ and LHRH-BinB_C_ for 48 h, Hs68 (**A**) and MCF-7 (**B**) cells were assessed the plasma membrane permeability by LDH release assay. LDH efflux (%) was determined by comparing the absorbance values of reaction mixtures from treated cells to those of untreated cells (low control) and cells treated with lysis solution (high control). The data are presented as the mean ± SD of three independent experiments, each containing three replicates. Statistical significance was analyzed using the *t*-test and annotated as follows: *** *p* ≤ 0.001, * *p* ≤ 0.05; ns, not significant (*p* > 0.05).

The plasma membrane damage mediated by BinB_C_ and LHRH-BinB_C_ was assessed by measuring the activity of cytoplasmic lactate dehydrogenase (LDH) cytoplasmic enzyme released to the extracellular medium of toxin-treated Hs68 and MCF-7 cells. After incubation of Hs68 and MCF-7 cell lines with different concentrations of BinB_C_, the amount of LDH efflux from either Hs68 or MCF-7 cells was relatively low compared with the untreated cells, although the LDH leakage was slightly higher in BinB_C_-treated Hs68 cells, suggesting that BinB_C_ does not induce noticeable damage to the membrane permeability of both Hs68 and MCF-7 cells (Figure 3A,B). On the other hand, the LDH efflux from the LHRH-BinB_C_-treated MCF-7 cells increased in a dose-dependent manner (Figure 3B). Moreover, when the protein concentration reached its maximum at 16 μM, there was a significant increase in LDH leakage from LHRH-BinB_C_-treated MCF-7 cells (Figure 3B), indicating that LHRH-BinB_C_ caused plasma membrane damage in MCF-7 cells.

### 2.4. LHRH-BinB_C_ Induces MCF-7 Cell Apoptosis by Activating Caspase-8

**Figure 4:**
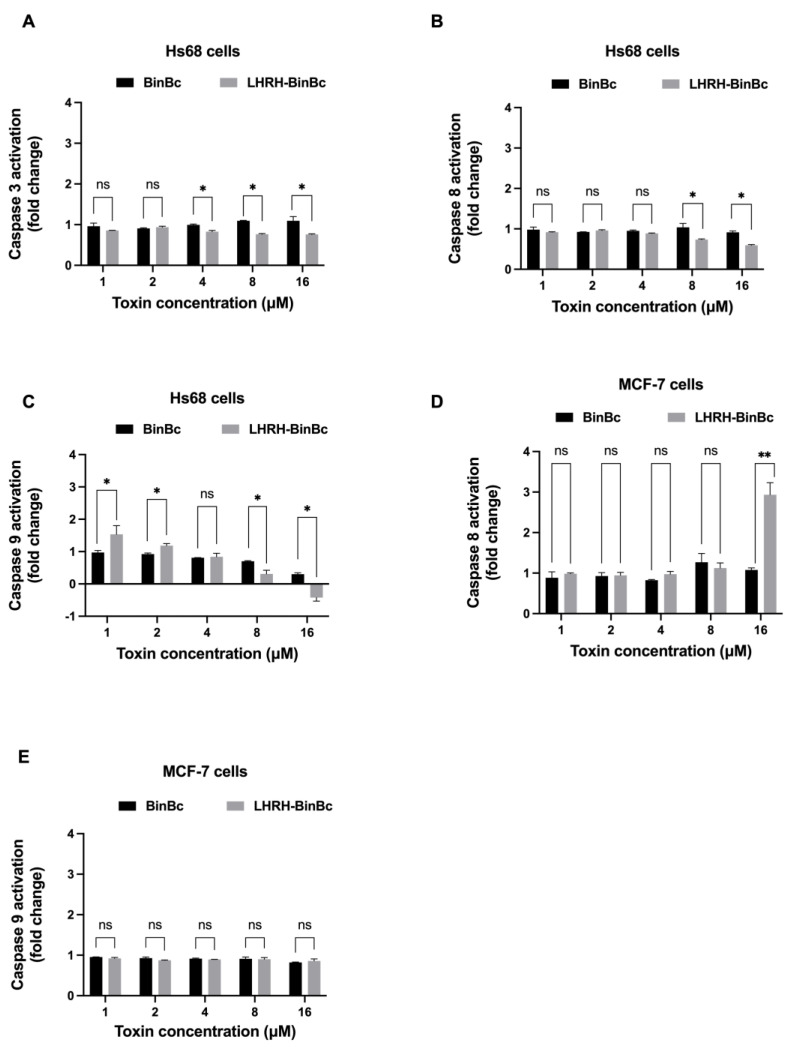
The activity of caspase-3, -8, and -9 in Hs68 cells (**A**–**C**) and caspase-8 and -9 in MCF-7 cells (**D**,**E**) after 48-h toxin exposure. Cells were treated with BinB_C_ and LHRH-BinB_C_ at 1, 2, 4, 8, and 16 μM, and the activity of caspase was measured using Caspase-3, -8, and -9 Multiplex Activity Assay. Readings obtained from treated cells were compared with measurements from control cells (untreated cells) and expressed as fold change. Each experiment was performed in duplicate. Statistical significance was analyzed using the *t*-test and annotated as follows: ** *p* ≤ 0.01, * *p* ≤ 0.05; ns, not significant (*p* > 0.05).

To investigate the cytotoxic mechanism of BinB_C_ and LHRH-BinB_C_ on Hs68 and MCF-7 cells, the activity of apoptosis-related proteins including caspase-3, caspase-8, and caspase-9 was determined upon exposure to the toxins as shown in Figure 4. The activity of caspase-3, -8, and -9 was not increased after treating Hs68 cells with LHRH-BinB_C_ at every tested concentration (1–16 μM) (Figure 4A–C), confirming their non-cytotoxic effect on normal cells as previously observed with the MTT and LDH release assays. As the MCF-7 cell line is caspase-3 deficient, only the activity of caspase-8 and caspase-9 was measured in this cell line [21]. Following treatment with BinB_C_ at every concentration, no increased activity of caspase-8 and -9 was observed (Figure 4D,E), suggesting the absence of apoptosis induction in BinB_C_-treated MCF-7 cells. After treating MCF-7 cells with LHRH-BinB_C_ at the highest tested concentration (16 μM), there was a significant increase in caspase-8 activity but no effect on caspase-9 activity (Figure 4D,E).

### 2.5. Protein Localization of LHRH-BinB_C_

**Figure 5:**
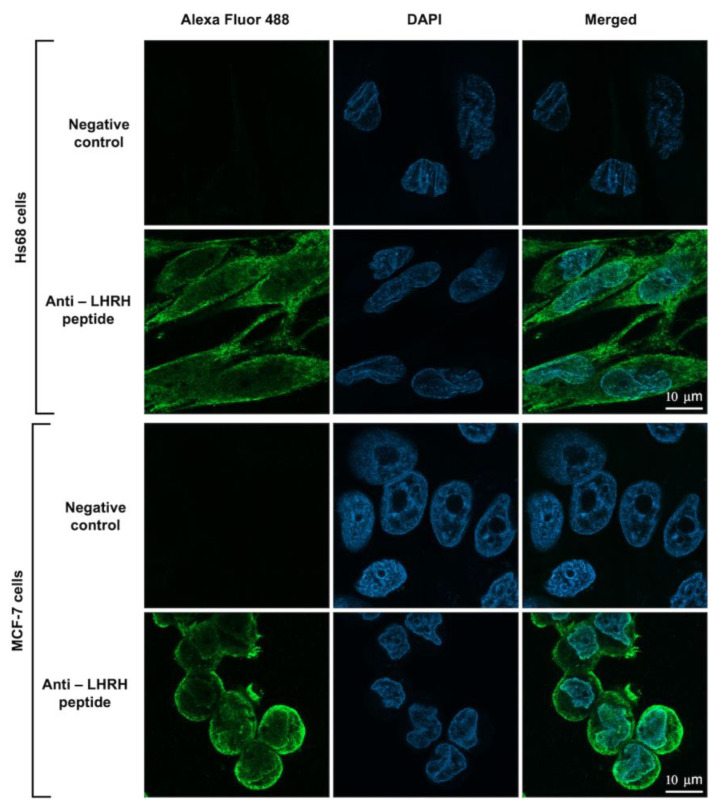
Internalization and localization of LHRH-BinB_C_ toxin in Hs68 and MCF-7 cells. The internalization and localization of LHRH-BinB_C_ in Hs68 and MCF-7 cells were investigated by using an immunofluorescence assay (IFA). Cells incubated with only the secondary antibody diluent without adding the primary antibody served as a negative control. LHRH-BinB_C_ (1 μM) was labelled with Alexa Fluor 488 (green) and the nucleus was stained with DAPI (blue). Images were taken with a confocal microscope (63× objective). The brightness and contrast of the images were adjusted to enhance their quality.

**Figure 6:**
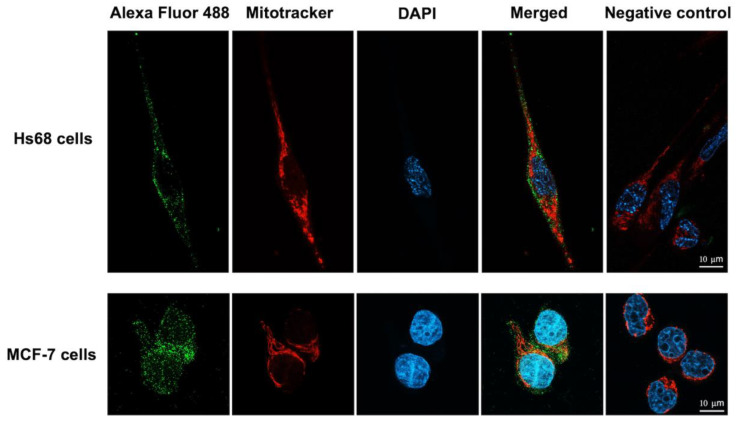
Colocalization analysis of LHRH-BinB_C_ toxin and mitochondria in Hs68 cells and MCF-7 cells. The colocalization between LHRH-BinB_C_ and mitochondria in Hs68 and MCF-7 cells was analyzed using immunofluorescence assay (IFA). Cells incubated with only the secondary antibody diluent without adding the primary antibody served as a negative control. LHRH-BinB_C_ (1 μM) was labelled with Alexa Fluor 488 (green), the mitochondria were stained with MitoTracker Red (red), and the nucleus was stained with DAPI (blue). Images were taken (in Z-stack and then compressed into 2D images with a maximum intensity projection) with a confocal microscope (63× objective). The brightness and contrast of the images were adjusted to enhance their quality. Three-dimensional reconstruction videos are provided in Videos S1 and S2.

To investigate whether the engineered LHRH-BinB_C_ protein could target the LHRH receptor present on the surface of MCF-7 cells, LHRH-BinB_C_ toxin at 1 μM, a sublethal concentration causing 20–30% inhibition of cell proliferation, was used to treat Hs68 and MCF-7 cells, and internalization and localization of LHRH-BinB_C_ toxin in both cells were then investigated by using immunofluorescence and confocal microscopy. The confocal images revealed that LHRH-BinB_C_ toxin was primarily found on the membrane surface of both Hs68 and MCF-7 cells, as indicated by green fluorescence (Figure 5). However, some green fluorescent signals of LHRH-BinB_C_ toxin were also detected inside the cytoplasm of both Hs68 and MCF-7 cells (Figure 5). The association of LHRH-BinB_C_ toxin with mitochondria was further explored by performing a colocalization assay (staining LHRH-BinB_C_ with a green fluorophore while staining mitochondria with a red fluorescent dye) (Figure 6). In this study, a plugin for ImageJ was used to determine Pearson’s correlation coefficient value (r). The value +1 represents a perfect correlation, while −1 represents a perfect anti-correlation. A value approaching 0 indicates a decreasing or nonexistent association. Pearson’s correlation coefficient values (r) for the colocalization of LHRH-BinB_C_ toxin with mitochondria in Hs68 and MCF-7 cells were 0.48 and 0.57, respectively, which are close to 0. Therefore, neither Hs68 nor MCF-7 cells demonstrated colocalization of LHRH-BinB_C_ toxin with mitochondria.

## 3. Discussion

Pore-forming toxins (PFTs) are the most common bacterial cytotoxic proteins that recognize and permeabilize cell membranes, leading to osmotic cell lysis and cell death [3]. Owing to these properties, they have a medical potential to destroy the target cells such as cancer cells. Among the pore-forming toxins, aerolysin-like PFTs constitute the main class of PFTs and offer high advantages as being specific to several types of cancer cells such as parasporin-family toxins [12]. Moreover, some aerolysin-like PFTs can be modified by conjugating with cell-targeting peptides (CTPs) so that they become selective in killing cancer cells [6,22]. Aerolysin-like PFTs including parasporins and Bin toxin produced by entomopathogenic bacteria *Bacillus thruringiensis* (Bt) and *Lysinibacillus sphaericus* (Ls), respectively, are attractive toxins that can be applied in cancer therapy due to their intrinsic properties in killing certain human cancer cells. Despite being less active in killing cancer cells than parasporins [14,15], Bin toxin from Ls could be engineered to selectively target cancer cells by adding a ligand moiety to specifically direct the toxin to certain types of binding sites on cancer cells. Our previous study showed that the truncated form of BinB consisting of only the aerolysin-like pore-forming domain or BinB_C_ was expressed as a soluble monomer and could interact and disrupt the membrane, giving a larger conductance and higher calcein release than those of the full-length BinB [13]. With its high membrane permeability, small size, and ease of large-scale purification, the BinB_C_ is thus a good candidate for engineering as a targeted cancer therapeutic agent.

Taking into account the favorable properties of LHRH peptide in drug delivery system, here, for the first time, we produced a fusion protein of BinB pore-forming domain (BinB_C_) and LHRH targeting peptide (LHRH-BinB_C_) via the peptide linker containing glycine and serine (GSG) to target the LHRH receptor-associated breast cancer cells. The flexible peptide linker containing glycine and serine (GSG) was introduced between LHRH and BinB_C_ to provide flexibility because glycine and serine are small molecules that help stabilize and maintain the linker structure in aqueous solution by forming hydrogen bonds with water molecules. The engineered LHRH-BinB_C_ protein was successfully expressed in *E. coli* as a soluble form with a high yield that could be easily purified to homogeneity. Due to its smaller size, LHRH-BinB_C_ fusion was expected to have better tumor penetration and reduce immunogenicity compared with other pore-forming immunotoxins [23,24].

Upon treatments with MCF-7 and Hs68 cells, the LHRH-BinB_C_ potently inhibited the proliferation of MCF-7 cells, giving an IC_50_ value of about 10.96 μM, while having little effect on Hs68 cells. In contrast, no inhibitory effect on the viability of MCF-7 and Hs68 cells was observed after treatment with BinB_C_. The LDH assay was used to indicate the permeability increase of the membrane as a consequence of the disruption of membrane integrity. In the absence of a time-interval assay in this study, LDH assay demonstrated a greater degree of LDH efflux from MCF-7 cells than that of Hs68 cells after 48 h of LHRH-BinB_C_ exposure, particularly at the maximum concentration (16 μM), suggesting the ability of LHRH-BinB_C_ to interact and form pores on the MCF-7 target membranes. We also conducted an experiment with [D-Lys^6^] LHRH, an LHRH agonist, to examine the impact of free LHRH on cell cytotoxicity. Our results demonstrated that [D-Lys^6^] LHRH was only cytotoxic to MCF-7 cells when utilized at the maximum tested concentration of around 320 μM, which is significantly higher than the IC_50_ of LHRH-BinB_C_ (10.96 μM). Hence, the cytotoxic effect of LHRH-BinB_C_ on MCF-7 cells might be mostly attributable to the pore-forming activity of BinB_C_. All of these in vitro cytotoxicity results show that fusing the BinB pore-forming domain with the LHRH targeting peptide at its N-terminus did not interfere with BinB_C_’s pore-forming activity, but rather increased cytotoxicity towards MCF-7 breast cancer cells. As a result, the LHRH peptide is highly effective in directing the BinB_C_ toxin to selectively target MCF-7 cancer cells.

Given the multiple ways a cell can die, overcoming apoptosis resistance or inducing apoptotic cell death would be the most effective technique for targeting cancer cells. In this study, apoptosis-related proteins from the caspase family were analyzed to assess whether LHRH-BinB_C_ induces apoptosis in MCF-7 cells compared to Hs68 cells. We initially focused on the LHRH-BinB_C_-mediated apoptosis as the cytotoxic mechanism based on previous findings that Bin toxin (specifically BinB subunit) induced cytotoxicity in various cancer cells, particularly the human nasopharyngeal carcinoma cells (HK1), and were also found associated with mitochondria of human liver cancer cells (HepG2), causing the cell apoptosis [14,15]. In addition, both caspase-3 and caspase-8 were elevated in HK1 treated with BinB [25], indicating that apoptosis induction is the cytotoxic mechanism underlying the cancer cell death upon Bin treatment. The caspase-cascade system plays an important role in the induction, transduction, and amplification of the intracellular apoptosis pathway [26]. Caspase-3, caspase-8, and caspase-9 are the caspase family’s key proteins in determining cell apoptosis or programmed cell death. Caspase-8 is a death receptor (extrinsic) apoptosis initiator caspase, whereas caspase-9 is a key initiator caspase required for apoptosis signaling via the mitochondrial (intrinsic) pathway. Caspase-3 is required for some morphological changes and biochemical events associated with the execution and completion of apoptosis after activation by initiator caspases [27,28,29]. It has been revealed that the MCF-7 cell line is caspase-3 deficient; nonetheless, the cells are still sensitive to cell death induction. It has been proposed that another executioner, caspase-7, could compensate for the role played by caspase-3 in the apoptosis cascade [21]. Only caspase-8 activity was increased in MCF-7 cells in response to LHRH-BinB_C_ treatment at high concentrations, whereas no caspase induction was observed in LHRH-BinB_C_-treated Hs68 cells. In addition, caspase-8 is implicated in suppressing a necrotic cell death pathway, known as necroptosis [30,31], suggesting that the extrinsic apoptosis pathway may play a role in the LHRH-BinB_C_-induced cell death in MCF-7 cells. To corroborate this conclusion, however, further investigation into the extrinsic apoptotic pathway induced by LHRH-BinB_C_ is required.

According to the previous study, the LHRH receptor has been shown to be overexpressed in A2780 ovarian carcinoma, MCF-7 breast cancer, and PC-3 prostate cancer cells but not in cells from other healthy organs [32]. It has been demonstrated that the binding between the LHRH peptide and LHRH receptor (LHRH-R) on the surface of cancer cells leads to the internalization of the toxin into the cells, possibly via receptor-mediated endocytosis [33]. To investigate whether the LHRH-BinB_C_ can interact and enter the cells, confocal microscopy was used to determine the internalization and localization of LHRH-BinB_C_ toxin in the cells. We found that LHRH-BinB_C_ toxin accumulated predominantly on the surface of both MCF-7 and Hs68 cells, suggesting that it binds to LHRH receptors (LHRH-R) on the plasma membrane of both cell lines. This suggests that the LHRH receptor may be somewhat expressed on the surface of Hs68 cells. However, whether Hs68 fibroblast cells express the LHRH receptor has yet to be determined. Moreover, some signals of LHRH-BinB_C_ toxin were also detected inside the cytoplasm of both cells. It should be noted that LHRH-BinB_C_ at a concentration of 1 μM employed in this experiment did not cause cytotoxicity in Hs68 cells, whereas the viability of MCF-7 cells was reduced by about 20–30% in response to the same concentration of LHRH-BinB_C_. Consequently, the observed interaction of LHRH-BinB_C_ with Hs68 cells under a confocal microscope may not be associated with cytotoxicity. BinB_C_ was excluded from the internalization and localization experiment due to non-cell cytotoxicity and minimal LDH release in BinB_C_-treated MCF-7 and Hs68 cells.

Mitochondria are cellular organelles known to play essential roles in energy metabolism, calcium homeostasis, immunity regulation and apoptosis. Mitochondrial dysfunctions, such as abnormalities in energy metabolism, increased transmembrane potential, and higher reactive oxygen species production, are frequently observed in cancer cells. Therefore, any bacterial toxins and other proteins targeting mitochondria are promising anticancer agents [34]. Thus, it is of interest to investigate whether the LHRH-BinB_C_ could associate with mitochondria, which would be a possible target for cancer therapy. We found no colocalization of the LHRH-BinB_C_ toxin and mitochondria in both MCF-7 and Hs68 cell lines. This finding suggests that the LHRH-BinB_C_ does not directly target mitochondria, which is consistent with the lack of caspase-9 activation, which has been associated with the intrinsic or mitochondrial-mediated apoptosis pathway. Our previous studies have demonstrated that the Bin toxin, especially the BinB subunit, induces cytotoxicity in various cancer cells, such as human nasopharyngeal carcinoma cells (HK1) and human liver cancer cells (HepG2) [14,15]. It was also found that BinB facilitates intracellular translocation of BinA in HepG2 cells, and the BinAB complex appears to associate with the mitochondria of the cells to cause apoptotic cell death [15]. A recent study reported that BinB potentially mediates the translocation of BinA into the cytoplasm of HK1 cells and associates with the mitochondria to trigger apoptotic events [25]. In contrast to the previous studies, we discovered that the pore-forming domain of BinB linked with the LHRH targeting peptide works primarily on the cell surface, with no need for internalization into the cytosol to cause cell cytotoxicity. BinB_C_’s pore-forming activity, guided by the specific binding of LHRH with LHRH-R, would result in changes in intracellular ions, which would then activate the apoptotic signaling pathway [35]. Other members of the aerolysin-like toxin family, such as aerolysin (*Aeromonas* species), lamprey immune protein (LIP), and parasporin-2 (PS2), have also been shown to induce apoptosis in their targeted cells [36,37,38,39]. Intriguingly, the apoptotic pathway induced by LHRH-BinB_C_ without the need for toxin internalization may circumvent several obstacles, such as a low tissue penetration rate, cytosol delivery defect, and toxin degradation in lysosomes prior to exerting their cytotoxic effect, making LHRH-BinB_C_ a promising candidate for development as an alternative cancer therapeutic agent.

## 4. Conclusions

We describe here, for the first time, an engineered pore-forming domain of BinB fused with the LHRH peptide (LHRH-BinB_C_) to selectively target breast cancer cells while causing no harm to normal cells. Our findings indicate that the engineered LHRH-BinB_C_ toxin has specific cytotoxicity against LHRH-positive cells in vitro (MCF-7 cells) and may induce cell death via apoptosis, making this engineered Bin protein a potential alternative for targeted breast cancer therapy.

## 5. Materials and Methods

### 5.1. Construction of BinB_C_ Fused with LHRH Targeting Peptide

A recombinant plasmid (pET28b-LHRH-BinB_C_) was constructed by fusing LHRH peptide (QHWSYGLRPG) and a linker (GSG) to the C-terminal domain or pore-forming domain of BinB (BinB_C_) protein using PCR-based site-directed mutagenesis (Non-QuikChange protocol) (Figure 1A). Mutagenic forward (5′ CAG CAT TGG AGC TAT GGC CTG CGC CCG GGC GGT AGC GGC ATT CCT CAA TTA CCC CAA ACA TCC TTA CTT GAG AAT ATT CCT GAG CCT ACT AGT C 3′) and reverse primers (5′ CAT ATG GCT GCC GCG CGG CAC C 3′) were phosphorylated at 5′ ends before ligation. The 5′ end of the forward primer included the LHRH peptide and linker sequences (underlined), while the reverse primer was designed to be upstream (reverse-complement) of the forward primer’s sequence. After PCR, linear PCR products (pET28b-LHRH-BinB_C_) were purified using a PCR Cleanup Kit (Geneaid column) and treated with *Dpn*I to remove the parental DNA before ligation to circularize the mutagenized plasmid. Following bacterial transformation, recombinant plasmids (pET28b-LHRH-BinB_C_) were isolated from the bacterial transformants and initially screened by restriction digestion analysis (*Nde*I and *Bam*HI) before DNA sequencing was used to validate the entire sequence (Macrogen, Seoul, Republic of Korea).

### 5.2. Protein Expression and Purification

For protein expression, the recombinant plasmid pET28b-LHRH-BinB_C_ was transformed into *E. coli* BL2(DE3)pLysS strain. The expression of His-tagged LHRH-BinB_C_ was then induced by adding 0.2 mM IPTG to an exponential growth culture and continuously growing at 18 °C for 5 h. The recombinant protein expression was analyzed by 12% SDS-PAGE and western blotting using HRP-conjugated anti-polyhistidine antibody [HIS-1] at a 1:5000 ratio. After verification, cells were harvested by centrifugation at 6000 rpm for 10 min at 4 °C. The harvested cells expressing His-LHRH-BinB_C_ were resuspended in an ice-cold lysis buffer (50 mM Tris-HCl and 200 mM NaCl, pH 8.0) and completely lysed by ultrasonication. The soluble and insoluble forms of the extracted protein were separated by centrifugation at 8000 rpm for 1 h at 4 °C. Supernatants were collected and filtered with a 0.45 μm syringe filter before performing Ni-NTA affinity chromatography. After equilibrating the HisTrap^TM^ Fast Flow column (prepacked with pre-charged Ni^2+^) with lysis buffer, the supernatant was loaded into the column. Nonspecific bound proteins were removed by washing columns with a buffer containing low concentrations of imidazole (25 and 50 mM). Then the bound target protein was eluted from the resin by using a buffer with increasing concentrations of imidazole (100 and 250 mM). All the unbound and eluted fractions were collected and analyzed by 12% SDS-PAGE. Following SDS-PAGE analysis, the fractions containing the purified recombinant protein (His-LHRH-BinB_C_) were pooled and concentrated by using a centrifugal filter unit (10 kDa cutoff, Amicon^®^). The concentrated recombinant protein was collected and purified again using gel filtration chromatography (Superdex 200 HR 10/30 column, GE Healthcare Life Science). UV absorption at 280 nm was used to check the concentration of the purified protein and eluted protein fractions from the column were collected and analyzed using SDS-PAGE.

In this study, a polyhistidine tag was fused to the LHRH-BinB_C_ protein, and the presence of the His_6_ tag may interfere with downstream applications or functions of the protein. Therefore, the His_6_ tag was removed by cleaving the protease site placed between the LHRH-BinB_C_ protein and the His6 tag, followed by separating the protein from the cleaved affinity tag. The complete digestion was done by incubating the purified His-LHRH-BinB_C_ protein with thrombin at 4 °C for 16 h, and the reaction was monitored for a protein band shift via SDS-PAGE analysis. After complete digestion, thrombin was inactivated by adding 1 mM phenylmethylsulfonyl fluoride (PMSF) into the mixture, and the cleaved protein was purified again using Ni-NTA affinity chromatography.

The expression and purification of BinB_C_ protein were prepared following protocols as described previously [13]. Briefly, BinB_C_ was cloned and expressed as a His_6_-tagged protein using *E. coli* BL21(DE3)pLysS as a host strain. The protein expressed as a soluble form was purified using Ni–NTA affinity and size-exclusion chromatography, following the same protocols as described previously for recombinant LHRH-BinB_C_.

### 5.3. Cell Culture and Cell Cytotoxicity Analysis

The breast cancer cell line MCF-7 (ATCC HTB22, Manassas, VA, USA) and human foreskin fibroblast cell line Hs68 (ATCC CRL-1635, Manassas, VA, USA) were acquired from the American Type Culture Collection (ATCC, Manassas, VA, USA). Hs68 and MCF-7 cells were cultured at 37 °C, 5% CO_2_, in DMEM (Gibco, Billings, MT, USA) and EMEM (ATCC, Manassas, VA, USA), both supplemented with 10% (v/v) heat-inactivated fetal bovine serum (FBS) (Gibco, Billings, MT, USA) and 1% penicillin/streptomycin (Gibco, Billings, MT, USA). The viability of Hs68 and MCF-7 cells was evaluated following treatment with toxins at several concentrations using buffer (Tris-HCl pH 9.0) as a negative control. The morphological alterations of Hs68 cells and MCF-7 cells after being treated with toxins for 48 h were observed under an inverted light microscope (Nikon Eclipse TS100, Melville, NY, USA). Moreover, the MTT assay was used to measure cell proliferation or cell cytotoxicity. Briefly, 1 × 10^4^ cells of Hs68 and MCF-7 cell lines were seeded into each well of a 96-well plate and cultured for 48 h. Different concentrations of toxins (1, 2, 4, 8, and 16 μM) were added to the corresponding well (three replicates), and the plate was incubated for 48 h. Then, 10 μL of MTT solution (Invitrogen, Carlsbad, CA, USA) at a final concentration of 5 mg/mL was added. After incubation for 4 h, 100 μL of dimethyl sulfoxide (DMSO, Lenexa, KS, USA) (Merck, Darmstadt, Germany) was added to each well to dissolve the formazan crystals in the cells before measuring the absorbance at 595 nm using Beckman Coulter DTX 880 Microplate Reader. The percentage of cell viability was determined by comparing the absorbance of each sample with that of the negative control, and the IC_50_ value was calculated as the test sample concentration required for 50% cell growth inhibition.

### 5.4. Lactate Dehydrogenase (LDH) Efflux Assay

To determine the plasma membrane damage of the cells as a result of protein toxin treatment, 1 × 10^4^ cells of Hs68 and MCF-7 cell lines were seeded into each well of a 96-well plate and cultured for 48 h. Cells were incubated with toxins (BinB_C_ and LHRH-BinB_C_) at 1, 2, 4, 8, and 16 μM for 48 h. After toxin incubation, lactate dehydrogenase (LDH, Tokyo, Japan) leakage was determined using LDH-Cytotoxicity Assay Kit II (Abcam, ab65393, Cambridge, MA, USA) as described in the manufacturer’s protocol. The absorbance was measured at 450 nm with the reference wavelength at 650 nm using a microplate reader (Biochrom EZ Read 2000, Cambridge, UK), and the experiments were done in triplicates. LDH efflux (%) was determined by comparing the absorbance values of reaction mixtures from treated cells to those of untreated cells (low control) and cells treated with lysis solution (high control).

### 5.5. Caspase-3, -8, and -9 Activity Assays

Cell apoptotic detection was determined using Caspase 3, Caspase 8, and Caspase 9 Multiplex Activity Assay Kit (Fluorometric) (ab219915) according to the manufacturer’s protocol. Briefly, 2 × 10^4^ cells of Hs68 and MCF-7 cell lines were seeded into the black wall and clear bottom of a 96-well plate and cultured for 48 h. Toxins (BinB_C_ and LHRH-BinB_C_) at different concentrations (1, 2, 4, 8, and 16 μM) were incubated with the cells for 48 h. Untreated cells were used as a control. After toxin incubation, fluorogenic indicators for caspase-3, caspase-8, and caspase-9 activity from the kit were added and incubated at room temperature in the dark for 1 h. Then the intensity of fluorescence was detected using a fluorescence microplate reader (TECAN, infinite 200Pro, Grödig, Austria) at the specific wavelengths (Caspase-3: Ex/Em = 535/620 nm, Caspase-8: Ex/Em = 490/525 nm and Caspase-9: Ex/Em = 370/450 nm). Caspase activation was evaluated by using fluorescence intensity to determine fold change between control and treated cells, and the experiments were performed in duplicate.

### 5.6. Toxin Internalization and Localization

The breast cancer cells (MCF-7) and human foreskin fibroblast cells (Hs68) were seeded on coverslips (1 × 10^5^ cells/well in 24 well plates) and incubated at 37 °C in a 5% CO_2_ incubator for 24 h. LHRH-BinB_C_ toxin at a sublethal concentration of 1 μM was then treated on the cells for 24 h. To determine the association of LHRH-BinB_C_ with the mitochondria in both MCF-7 and Hs68 cells, the treated cells were washed with 1 × PBS buffer pH 7.4 and then incubated with 500 nM MitoTracker Red CMXRos dye (Invitrogen/Molecular Probes, Eugene, OR, USA) at room temperature for 30 min. Cells were then fixed and permeabilized following standard protocol. The cells were incubated with a primary antibody specific to LHRH peptide at a 1:5000 ratio (rabbit polyclonal; AB1567, Chemicon, Temecula, CA, USA) followed by a secondary antibody conjugated with Alexa Fluor 488 at a 1:5000 ratio (goat anti-rabbit IgG H&L (Alexa Fluor^®^ 488, Waltham, MA, USA); AB150077, Invitrogen, Thermo Fisher Scientific, Inc., Waltham, MA, USA). Cells incubated with only the secondary antibody diluent without adding the primary antibody served as a negative control. Fixed cells were mounted with a mounting medium before observing under a confocal laser scanning microscope (Carl Zeiss LSM800). For mounting cells onto microscope slides, 2 μL of antifade mounting reagent was dropped on a cleaned glass slide, and the coverslip with the fixed cells was then carefully placed (the cover slip facing down to the glass slide) onto the glass slide.

### 5.7. Statistical Analysis

After completing three independent replicates, the data are presented as mean ± standard deviation (SD). Statistical analysis was performed by using *t*-test and GraphPad Prism Version 9.5.0 (525). Statistical significance was annotated as follows: *** *p* ≤ 0.001, ** *p* ≤ 0.01, * *p* ≤ 0.05; ns, not significant (*p* > 0.05).

## Data Availability

The data presented in this study are available within the manuscript and supplementary information files.

## Supplementary Materials

The following supporting information can be downloaded at: https://www.mdpi.com/article/10.3390/toxins15040297/s1, Video S1: 3D reconstruction video of colocalization analysis of LHRH-BinB_C_ toxin and mitochondria in Hs68 cells; Video S2: 3D reconstruction video of colocalization analysis of LHRH-BinB_C_ toxin and mitochondria in MCF-7 cells.
